# NEW CONSUMER-REPORTED QUALITY INDICATORS FOR HOME AND COMMUNITY-BASED SERVICES: THE NCI-AD SURVEY

**DOI:** 10.1093/geroni/igae098.1939

**Published:** 2024-12-31

**Authors:** Tetyana Shippee, Regina Shih

**Affiliations:** Chair; Discussant

## Abstract

This symposium will introduce new consumer-reported quality indicators for home and community-based services (HCBS). The HCBS Quality Measures Set released by the Centers for Medicare and Medicaid Services includes few consumer-reported measures, most of which are not psychometrically validated or widely applicable. The National Core Indicators- Aging and Disability Survey (NCI-AD) is an underutilized source of consumer-reported data for measuring HCBS quality. This symposium includes 4 presentations. [1] The first presentation provides an overview of the NCI-AD database for researchers and policy-makers, including new features/survey tools added to more recent survey waves. [2] The second presentation introduces five new quality indicators (from 22 consumer-reported survey items) developed using exploratory and confirmatory factor analyses of the NCI-AD data. These indicators include staff quality, environmental safety, community inclusion, service satisfaction, and service decision-making; each with good reliability and validity (Cronbach’s alpha, 0.78-0.89). [3] The third presentation demonstrates measurement of service-specific unmet needs as a measure of consumer-reported quality for 6 commonly used HCBS, and how these differ between consumers with versus without dementia. [4] The fourth presentation discusses state-level factors as correlates of consumer-reported service-specific unmet needs, with emphasis on a higher proportion of Medicare managed care beneficiaries in a state as a predictor of greater unmet needs for some HCBS measures. Collectively, the four projects introduce new consumer-reported quality indicators for HCBS and equip the attendees with knowledge to measure consumer-reported quality in HCBS, including for older adults with dementia.

